# HOGA1 Suppresses Renal Cell Carcinoma Growth via Inhibiting the Wnt/β‐Catenin Signalling Pathway

**DOI:** 10.1111/jcmm.70490

**Published:** 2025-03-18

**Authors:** Congmin Wang, Yu Liu, Ying Tan, Fuyi Xu, Mingyao Wang, Yiming Tang, Guofeng Nie, Xiaodong Chi, Zhaowei Xu, Yuxue Xu, Baijiao An, Geng Tian, Donglai Qi, Cuifang Yao

**Affiliations:** ^1^ School of Pharmacy Binzhou Medical University Yantai China; ^2^ Shandong Technology Innovation Center of Molecular Targeting and Intelligent Diagnosis and Treatment Binzhou Medical University Yantai China; ^3^ The Second School of Clinical Medicine Binzhou Medical University Yantai China; ^4^ The First School of Clinical Medicine Binzhou Medical University Yantai China

**Keywords:** BXD, clear cell renal cell carcinoma (ccRCC), HOG, HOGA1, Wnt/β‐catenin

## Abstract

Changes in hydroxyproline metabolism are reported to promote tumorigenesis. HOGA1 is a useful marker for diagnosing primary hyperoxaluria 3, catalysing the final step of mitochondrial hydroxyproline metabolism from 4‐hydroxy‐2‐oxoglutarate (HOG) to glyoxylate and pyruvate; however, its specific mechanism in RCC remains unclear. This study investigated the role of HOGA1 in the pathogenesis of ccRCC. The results showed that HOGA1 was decreased significantly in tumour tissues, with this low expression associated with a poor prognosis in patients with ccRCC. QTL mapping showed that *Hoga1* was *cis*‐regulated. Gene enrichment analyses showed that *Hoga1* co‐expressed genes were enriched in the Wnt/β‐catenin signalling pathway. Furthermore, in vitro and in vivo assays demonstrated that HOGA1 significantly inhibited the proliferation, invasion and migration of renal carcinoma cells via the Wnt/β‐catenin–c‐Myc/CyclinD1 axis, probably via regulating the level of HOG. In conclusion, this study demonstrates that HOGA1 has a tumour suppressor role by inhibiting the Wnt/β‐catenin signalling pathway. This finding provides new insights into the function of HOGA1 in ccRCC.

**Abbreviations**
ccRCC: clear cell renal cell carcinoma
GSK‐3β: glycogen synthase kinase‐3 beta
GTEx: genotype‐tissue expression
HOG: 4‐hydroxy‐2‐oxoglutarate
HOGA1: 4‐hydroxy‐2‐oxoglutarate aldolase
PH3: primary hyperoxaluria type 3
RCC: renal cell carcinoma
RI: recombinant inbred

## 1 Introduction

Renal cell carcinoma (RCC) is a common and deadly urological malignancy with a worldwide estimate of approximately 430,000 new cases and 179,000 deaths in 2020 (Global Cancer Observatory) [1, 2, 3]. The most common form of RCC is the histological subtype called clear cell RCC (ccRCC) which accounts for 70%–80% of all cases [4]. Depending on the extent and state of the disease, the 5‐year survival rate for patients with RCC ranges from 20% to 95% [5]. However, the prognosis for patients with metastatic ccRCC is poor, with a 5‐year survival rate under 5%, due to their high risk of metastasis and intrinsic resistance to radiotherapy and chemotherapy [6, 7]. There is therefore a need to better understand the mechanisms underlying the pathogenesis of ccRCC and to identify better therapeutic targets.

Hydroxyproline is a nonessential amino acid found in collagen and a few other extracellular animal proteins. Various abnormalities of hydroxyproline metabolism have been found to play key roles in the pathophysiology and pathogenesis of different diseases. Elevated levels of hydroxyproline have been reported to be associated with increased collagen deposition in the tumour microenvironment, promoting tumour growth and invasion [8, 9]. Additionally, the metabolic pathways involving hydroxyproline may affect cellular signalling and the response to hypoxia, further linking hydroxyproline metabolism to cancer development and progression [10, 11, 12]. However, little is known about the role of hydroxyproline metabolism in the pathogenesis of ccRCC.

HOGA1 (4‐hydroxy‐2‐oxoglutarate aldolase, formerly known as DHDPSL) is a mitochondrial protein that catalyses the final step of mitochondrial hydroxyproline metabolism, converting 4‐hydroxy‐2‐oxoglutarate to glyoxylate and pyruvate [13, 14]. Loss‐of‐function mutations of HOGA1 in humans cause primary hyperoxaluria type 3 (PH3), a rare autosomal recessive disease characterised by excessive production of oxalate and kidney stones [15, 16]. While HOGA1 serves as a valuable diagnostic biomarker for PH3, its involvement in other diseases remains underexplored. A study reported that patients with HOGA1 gene mutations are at risk of forming renal cysts as a result of deposition of crystals or stones that lead to the development of tubular dilatation and activation of inflammatory vesicles [17]. Furthermore, a proteogenomic study on pancreatic ductal adenocarcinoma cancer showed that loss of HOGA1 promoted the proliferation of cancer cells by regulating the cell cycle [18]. A study by Li et al. [19] also indicated that HOGA1 may be a biomarker to predict ccRCC patient outcomes, although no mechanism for this association was proposed. Consequently, HOGA1 may play a significant role in various diseases, particularly those related to the kidneys, including renal cancer. A review of existing literature highlights that one of the primary phenotypes observed in PH3 patients is the formation of kidney stones, which has been associated with an elevated risk of kidney cancer [20, 21]. Based on the above findings, we speculate that HOGA1 may be a key protein in the regulation of renal cancer.

In this study, we found that low‐expression HOGA1 in ccRCC tissues was associated with pathological grading and poor prognosis of ccRCC patients. Overexpression of HOGA1 effectively inhibited the proliferation and migration of ccRCC cells. In addition, we explored the regulation mode of HOGA1 expression through genome‐wide association studies, and using a systems genetic approach delineated the underlying molecular mechanisms in transcriptome data from BXD RI mice. Further study showed that HOGA1 has a tumour suppressor role by inhibiting the Wnt/β‐catenin signalling pathway via regulating the level of its substrate HOG.

## 2 Material and Methods

### 2.1 Tissue Samples

The project was approved by the Human Research Ethics Committee of Binzhou Medical College (Approval Code: 2021–202) and was conducted in accordance with the 1975 Declaration of Helsinki. After the patients had provided their written, informed consent to obtain tissue samples, all the ccRCC tissue samples were obtained from Yuhuangding Hospital from 2018 to 2020 (Table S1). Five pairs of sections were used for proteomic analysis, and the other six were used for Western blotting, real‐time polymerase chain reaction (RT‐PCR) and immunohistochemistry (IHC).

### 2.2 Protein Digestion

The protocol was similar to that described in our previous work [22]. Briefly, the tissue samples were suspended and homogenised in lysis buffer (9 mol/L urea, 20 m mol/L HEPES) containing a proteinase inhibitor cocktail (Thermo, A32961). After sonication, the samples were centrifuged at 4°C for 10 min at 12,000 × g and the supernatant was collected. A 20 μg aliquot of the total protein was diluted in 100 μL of digestion buffer (6 mol/L urea and 100 mmol/L TEAB), followed by the addition of 10 μL of 50 mmol/L DTT reduction solution, incubation of the sample at 50°C for 15 min and then the addition of 10 μL of 50 mmol/L IAA for alkylation in the dark for 15 min at room temperature. The samples were then diluted four times with digestion buffer (50 mM NH_4_HCO_3_, pH 8.0) and digested overnight at 37°C with trypsin (Thermo, ADV5111) at a final concentration of 5% (w/w). The reaction was stopped by diluting the sample 1:1 with trifluoroacetic acid (TFA) in acetonitrile (ACN) and Milli‐Q water (1/5/94 v/v). Finally, the peptides were desalted using Pierce C18 Spin columns (Thermo, SC2003) and dried completely in a vacuum centrifuge.

### 2.3 LC–MS/MS Analysis

The peptide analyses were performed using a Q Exactive Plus Orbitrap mass spectrometer (Thermo Fisher Scientific, Waltham, MA, USA), equipped with a nano‐electrospray ion source. The proteomics raw data were analysed using Maxquant (version 2.0.0.1) based on the Uniprot *Homo sapiens* database (April 2021).

### 2.4 BXD Mice Kidney Gene Expression Data

The data set was obtained from the Genenetwork website (https://www.genenetwork.org/) [23], which can be accessed publicly [24]. It includes detailed data from the ‘BXD Family’ group, ‘Kidney mRNA’ type and the ‘Mouse kidney M430v2 sex Balanced (Aug06) RMA’ data set, with the Record ID: Hoga1. The BXD mice were obtained from UTHSC and the Jackson Laboratory and housed before sacrifice at UTHSC, Harvard/BIDMC (Glenn Rosen), the University of Memphis (Douglas Matthews) or the Jackson Laboratory. The expression data were then renormalised using a modified *Z*‐score as described in a previous publication [25]. RMAs were first transformed into log_2_ values, and the data of every single array were then converted to a *Z*‐score, multiplied by 2 and a value of 8 was added.

### 2.5 *Hoga1* Genetic Variants and Expression QTL (eQTL) Mapping

The genetic variants of *Hoga1* between the parental strains B6 and D2 were determined using the mouse genome variants querying site of the mouse genome project (https://www.sanger.ac.uk/science/data/mouse‐genomes‐project) [26]. eQTL mapping of *Hoga1* gene expression was performed using WebQTL. The QTL confidence interval was estimated using the conventional 1.5 LOD drop‐off interval, in which LOD = LRS/4.61 [27].

### 2.6 Gene Coexpression Analysis

Gene coexpression network analysis has been used widely to investigate key genes in a gene set. Based on the Pearson correlation coefficient, we identified *Hoga1*‐related genes in the transcriptomic data of the BXD mouse kidney, genes with *p*‐values < 0.05 considered to be significantly associated with *Hoga1*.

### 2.7 Gene Set Enrichment Analysis

Genes with a statistically significant genetic correlation with *Hoga1* were selected and uploaded to Webgestalt (http://www.webgestalt.org/) for gene enrichment analysis. The *p*‐values generated from the hypergeometric test were corrected using the Benjamini and Hochberg method.

### 2.8 Cell Culture

Cell lines 786‐O, 769‐P and HEK293T were purchased from ATCC and were authenticated by short‐tandem repeat profiling. At 37°C, all cell lines were cultured in RPMI medium 1640 or DMEM medium with 5% CO_2_. The medium was supplemented with 10% fetal bovine serum (FBS) and 1% penicillin–streptomycin.

### 2.9 Western Blot

SDS‐PAGE was performed following the measurement of protein concentration, after which the sample was transferred to the PVDF membrane postseparation. The PVDF film was sealed with 5% fat‐free milk powder for 1 h at room temperature and then sealed overnight with the following antibodies at 4°C: rabbit anti‐HOGA1 (NOVUS, NBP3‐06562), rabbit anti‐GSK‐3β (CST, 12456S), rabbit anti‐β‐catenin (CST, 8480S), rabbit anti‐phospho‐β‐catenin (Ser33/37/Thr41) (CST, 9561 T), mouse β‐ACTIN (GenScript, A700702), rabbit anti‐CyclinD1 (Proteintech, 60,186‐1‐Ig) and rabbit anti‐c‐Myc (Wanleibio, WL01781). The primary antibody was recovered, and the PVDF membrane was washed with TBST for 10 min. The PVDF membrane was incubated with the appropriate secondary antibody for 1 h at room temperature. Finally, the bands were detected using the ECL chemical substrate luminescence detection kit.

### 2.10 CCK‐8

Transfected HOGA1 cells were inoculated in 96‐well plates (2 × 10^3^ cells per well) for 24 h. After 24 h, CCK‐8 reagent (10 μL) (Bimake, B34304) was added to each well and incubated for 2 h at 37°C in the incubator. Then OD450 was measured with a microplate reader.

### 2.11 Wound‐Healing Assay

Wound‐healing assay was used to determine the cell migration ability. Briefly, approximately 1 × 10^6^ cells were seeded in six‐well plates at equal densities and grew to 70%~80% confluency. Then, artificial gaps were generated by a 1 mL sterile pipette tip after transfection with the corresponding plasmids. Wounded areas were marked and photographed under a microscope at 0 and 24 h.

### 2.12 Transwell Analysis

For migration assay, 5 × 10^4^ cells were inoculated into a transwell (Corning, 353,097) superlayer with serum‐free 1640 media. The lower layer of the transwell was filled with 1640 medium with 10% FBS, and cells were cultured for 24 h. For invasion assay, the upper transwell chamber was precoated with Matrigel (BD, 354234), then 1 × 10^5^ cells were seeded in the upper transwell chamber and cells were cultured for 24 h. After culture, crystal violet staining was performed to observe cell migration and invasion.

### 2.13 IHC

HOGA1 expression was detected by IHC in ccRCC and adjacent nontumour tissues. The paraffin section of Kidney tissues was heated at 60°C for 2 h, then deparaffinised with gradient ethanol and incubated in boiling citrate buffer (0.01 mol/L, pH 6.0) for 10 min to accomplish the antigen retrieval. After being treated with 3% hydrogen peroxide for 15 min, the samples were blocked with bovine serum albumin for 1 h at 37°C and incubated in the HOGA1 antibody (NOVUS, NBP3‐06562, 1:100) and Ki67 (CST, 9449#, 1:200) overnight at 4°C. Finally, the DAB Horseradish Peroxidase Colour Development Kit was deployed to detect the immunoreactivity.

### 2.14 RT‐PCR

RNA was isolated using TRIzol reagent (R401‐01, Vazyme) per the manufacturer's protocol. RNA reverse transcription of purified RNA (1 μg) was performed using the HiScript III 1st Strand cDNA Synthesis Kit (+gDNA wiper) (R312‐01, Vazyme). The primer sequences are as follows: β‐ACTIN‐F: 5′‐GCCGACAGGATGCAGAAGGAGATCA‐3′; β‐ACTIN‐R: 5′‐AAGCATTTGCGGTGGACGATGGA‐3′; HOGA1‐F: 5′‐TTCCTTTCCTGACCAGCAGTG‐3′; HOGA1‐R: 5′‐GAGAGAGATCAGCAACCTTGG‐3′. The relative gene expression was calculated using the 2^−ΔΔCT^ method.

### 2.15 Xenograft Tumour Assays

Male, 4‐week‐old NOD SCID mice were selected, and the experimental mice were bought and placed in an SPF environment for adaptive observation for a week before the formal experiment began. For subcutaneous xenografts, 8 × 10^6^ cells in 100 μL PBS were injected subcutaneously into mice. Tumours were measured every 2 days after injection, and the tumour volume was calculated according to the formula: tumour volume (mm^3^) = (length × width^2^)/2. When the tumour grew to 1500 mm^3^, the mice were sacrificed and the tumours were taken for follow‐up experiments.

### 2.16 Statistical Analysis

Data were analysed with GraphPad Prism software. The results were presented as the mean ± SD. Migration, invasion and immunohistochemistry were quantified using Image J. Comparisons between two groups were evaluated using the two‐tailed Student's *t*‐test to determine significant *p*‐values. *p < 0.05, **p < 0.01, ***p < 0.001 and ****p < 0.0001 were considered statistically significant.

## 3 Results

### 3.1 Low HOGA1 Expression is a Poor Indicator in ccRCC Patients

**FIGURE 1:**
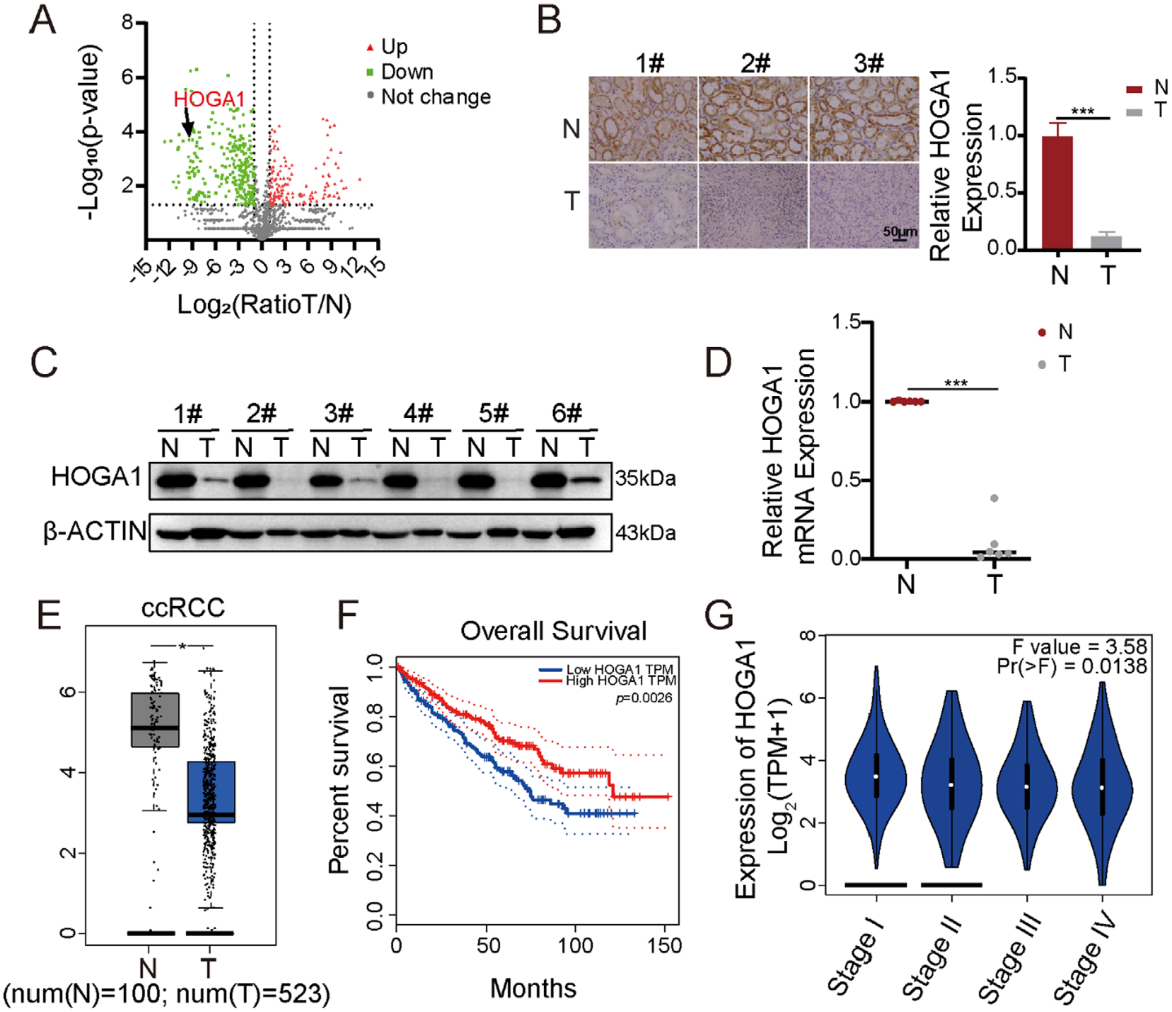
HOGA1 expression in ccRCC tissues and correlations to survival. (A) Volcano plot graph showing the differential expressed proteins in the quantitative analysis. The −log_10_ (*p*‐value) was plotted against the log_2_ (ratio T/N). N represented adjacent nontumour tissues. T represented tumour tissues. The upregulated proteins in ccRCC tissues were marked with red dots and the downregulated proteins in ccRCC tissues with green dots. (B) Relative HOGA1 expression was detected by IHC between ccRCC tumours and adjacent nontumour kidney tissues (*n* = 6, scale bars, 50 μm). (C) Protein levels of HOGA1 in ccRCC tumours and matched adjacent noncancer tissues were analysed by western blot (*n* = 6). (D) mRNA levels of HOGA1 in ccRCC tumours and matched adjacent noncancer tissues were analysed by RT‐PCR (*n* = 6). (E) The GEPIA database showed HOGA1 low expression in ccRCC compared to normal tissues (normal, *n* = 100; ccRCC, *n* = 523) cohorts. (F) Low‐level HOGA1 expression correlates with poor survival outcomes in ccRCC (high, *n* = 258; low, *n* = 258) cohorts based on survival analysis. (G) HOGA1 is downregulated with ccRCC grade increase based on the GEPIA database. *p < 0.05 and ***p < 0.001.

To find the metabolic enzymes that play important roles in the development and progression of ccRCC, we performed quantitative proteomic detection. As shown in Table S2, a total of 2076 proteins were identified and quantified. A volcano plot was generated to show the differential expression distribution (Figure 1A). We used the screening criteria of *p*‐value ≤ 0.05 and |Log_2_FoldChange| ≥ 1 to define differentially expressed genes, where *p*‐value ≤ 0.05 and Log_2_FoldChange ≥ 1 were upregulated genes, and *p*‐value ≤ 0.05 and Log_2_FoldChange ≤ −1 were downregulated genes. Of these 2076 proteins, 409 had significant differential expression between ccRCC tissues and adjacent nontumour tissues (*p* < 0.05, Table S3), with 134 significantly upregulated and 275 downregulated in the ccRCC samples. Among these genes, HOGA1 was significantly downregulated. Additionally, through a literature review and screening, we identified HOGA1 as a potential candidate. Our proteomic results indicated low expression of HOGA1 in ccRCC tissues, which was consistent with a previous report of mass spectrometric data [28]. This finding was further validated at the protein and transcriptional levels in tissues from six ccRCC patients using IHC, Western blotting and RT‐PCR (Figures 1B–D, S1A). The GEPIA database also showed that HOGA1 expression levels were downregulated in ccRCC tissues compared with those measured in nontumour tissues (Figure 1E). Moreover, low levels of HOGA1 expression were associated with significantly worse overall survival (Figure 1F), with HOGA1 expression decreasing with increasing tumour grade (Figure 1G). Additionally, we compared HOGA1 expression with tumour grade in 11 pairs of clinical renal cancer samples and found that six pairs were Grade I, while the remaining five were Grade II, further suggesting that higher tumour grade is associated with lower HOGA1 expression (Figure S1B). Taken together, these results indicated that HOGA1 may play an antioncogene role in ccRCC.

### 3.2 Overexpression of HOGA1 Suppresses Proliferation and Metastasis of ccRCC Cells In Vitro

**FIGURE 2:**
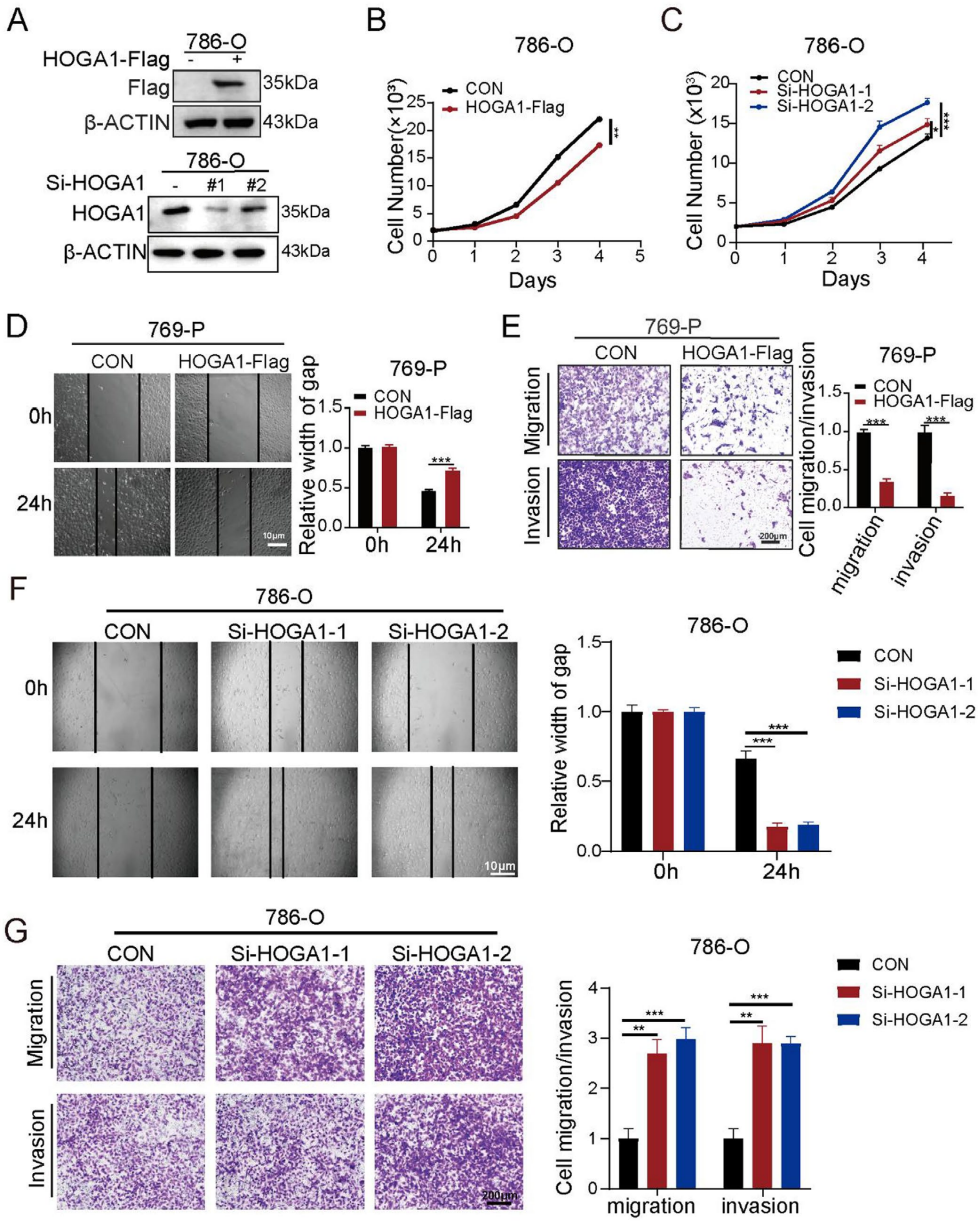
HOGA1 inhibits cell proliferation and migration in ccRCC cells. (A) Western blot validated the expression of HOGA1 and HOGA1 siRNA transfection in 786‐O cells. (B‐C) CCK‐8 assay was performed to examine the effect of HOGA1 overexpression and HOGA1 knockdown on cell viability in 786‐O cells. (D) Wound‐healing assay determined the migratory distances of HOGA1 overexpression and control. Scale bars, 10 μm. (E) The effect of HOGA1 overexpression on cell migration and invasion was evaluated by transwell assay. Scale bars, 200 μm. (F) Wound‐healing assay determined the migratory distances of HOGA1 knockdown and control. Scale bars, 10 μm. (G) The effect of HOGA1 knockdown on cell migration and invasion was evaluated by transwell assay. Scale bars, 200 μm. *p < 0.05, **p < 0.01 and ***p < 0.001.

To investigate the biological function of HOGA1 in ccRCC cells, HOGA1 was overexpressed and knocked down in 786‐O and 769‐P cells (Figures 2A, S2A). Cell proliferation of 786‐O and 769‐P with HOGA1 overexpression and knocked‐down HOGA1 was measured using the CCK‐8 assay (Figures 2B,C, S2B,C), while the wound‐healing assay (Figures 2D,F, S2D,F) and cell migration and invasion experiments (Figures 2E,G, S2E,G) were used to detect the effect of HOGA1 overexpression and knock down on the metastatic ability of the two types of cells. Our results verified that HOGA1 overexpression significantly suppressed growth compared with that in control cells and that the number of 786‐O and 769‐P cells that migrated and invaded after HOGA1 overexpression was also reduced significantly. These results demonstrated that HOGA1 has a potential inhibitory role in ccRCC cell growth in vitro.

### 3.3 System Genetics Analysis of *Hoga1* in BXD Mice Strains

Systems genetics analysis was then performed to determine the regulation of *Hoga1* expression. Gene expression levels of *Hoga1* in kidney tissue across the 54 BXD mouse strains and their corresponding parental strains were examined. There was only one probe set (1450098) in the Affymetrix Mouse Gene 2.0 ST array that targeted the proximal and mid 3’ UTR of the *Hoga1* gene.

**FIGURE 3:**
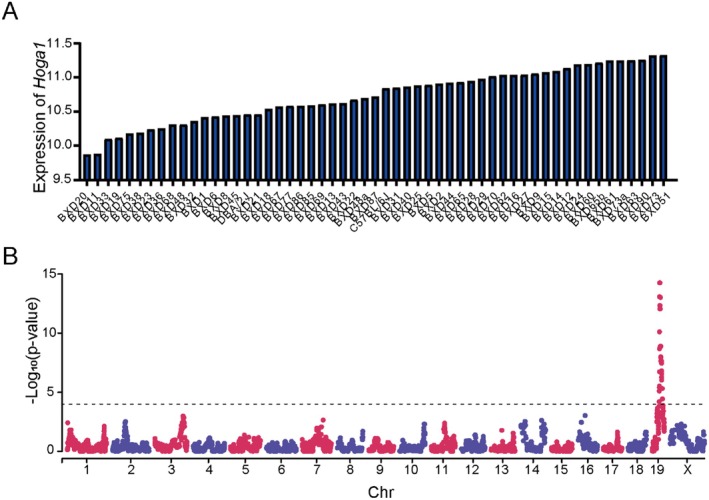
System genetics analysis of *Hoga1* in BXD mice strains. (A) Variable expression levels of *Hoga1* in the kidney tissue. The x‐axis denotes the strain, while the y‐axis denotes the mean expression given as log_2_. (B) Manhattan plot of genome‐wide *Hoga1*‐regulated genomic loci. The x‐axis denotes a position on the mouse genome, in megabases (Mb), while the y‐axis gives the ‐log_10_ (*p*‐value), a measurement of the linkage between *Hoga1* expression and genomic region. The dashed line indicates significant genome‐wide thresholds (*p* = 1 × 10^−4^).

The average expression of *Hoga1* across all BXD strains was 10.73 ± 0.02 [log_2_ scale, mean ± standard error of the mean (SEM)]. As shown in Figure 3A, BXD20 and BXD51 mice had the lowest and highest expression values for *Hoga1*, respectively. To identify the sequence variants that affected *Hoga1* expression in the mouse kidney, we performed simple interval mapping for *Hoga1* across the mouse genome. One significant expression QTL (eQTL) was identified, which encompassed a 4.5 Mb genomic region from 38 to 42.5 Mb. Based on the physical location of *Hoga1* on chromosome 19 at 42.07 Mb (Figure 3B), we defined the locus as a *cis*‐eQTL. This suggested that variation in *Hoga1* expression in BXD mice is modulated by alterations in its own sequence.

### 3.4 HOGA1 Inhibits Wnt/β‐Catenin Signalling Pathway

**FIGURE 4:**
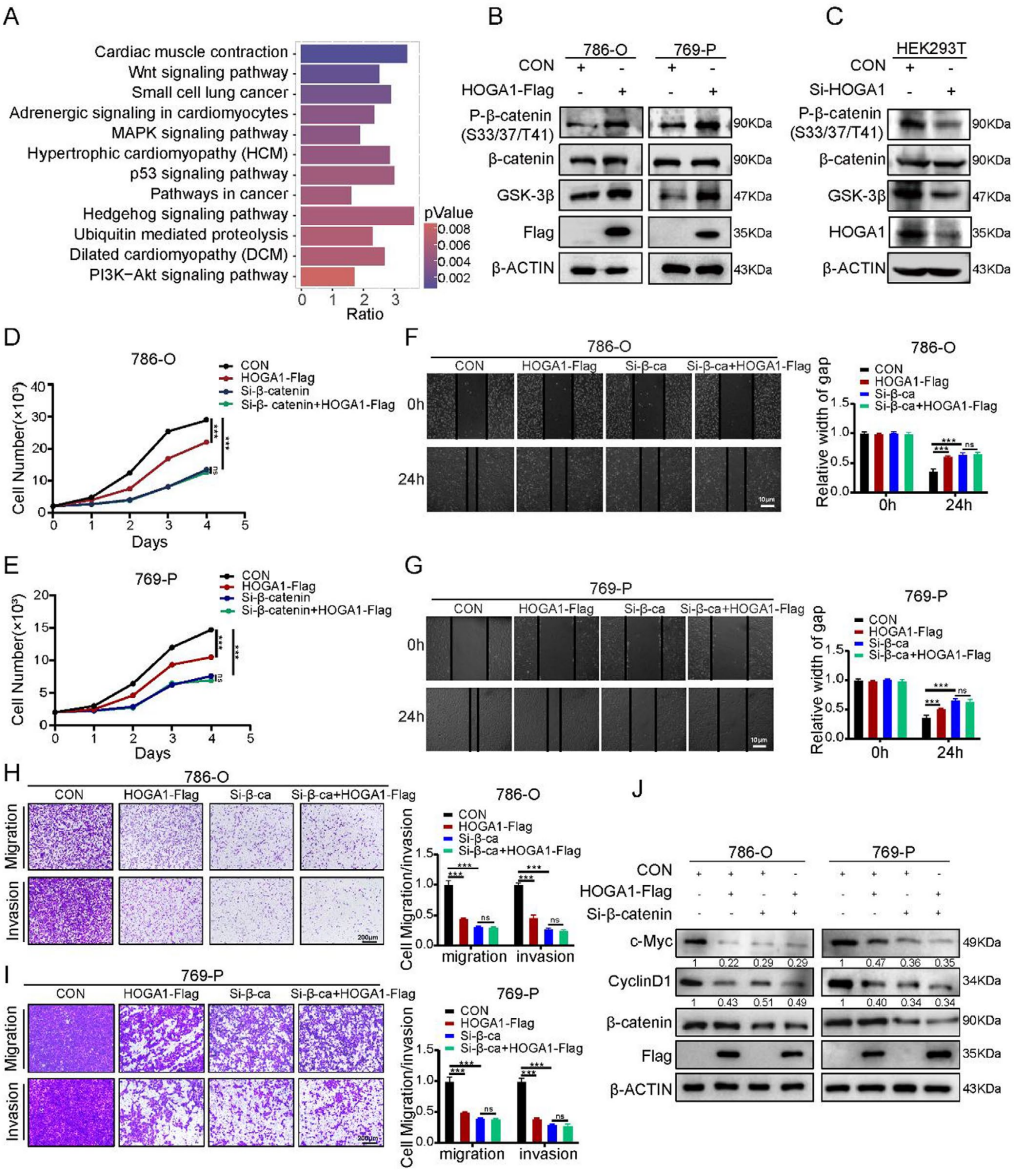
HOGA1 inhibits the Wnt/β‐catenin signalling pathway. (A) KEGG analysed the HOGA1 correlated genes. The x‐axis denotes the enrichment ratio and the y‐axis denotes the pathways. The colour represents the *p*‐value. (B) Effect of overexpression of HOGA1 on Wnt/β‐catenin signalling pathway. (C) Effect of knocking down of HOGA1 on Wnt signalling pathway. (D and E) Cell viability was evaluated with CCK‐8 assay to examine the effect of HOGA1 overexpression and Si‐β‐catenin. (F and G) Wound‐healing assay was conducted to analyse the metastasis of 786‐O and 769‐P cells with the treatment with Si‐β‐catenin (Si‐β‐ca), and overexpression of HOGA1. Scale bars: 10 μm. (H and I) Transwell migration and invasion assays were performed to analyse the metastasis as well as invasion of 786‐O and 769‐P cells with the treatment with Si‐β‐catenin (Si‐β‐ca), and overexpression of HOGA1. Scale bars: 200 μm. (J) Western blot validated the differential gene expression related to the Wnt/β‐catenin signalling pathway in 786‐O and 769‐P cells with Si‐β‐catenin (Si‐β‐ca), and overexpression of HOGA1. ns: Not significant, ***p < 0.001.

To understand the functional role of HOGA1 in ccRCC, we performed a gene set enrichment analysis using the top 2000 *Hoga1*‐coexpressed genes (*p* < 0.05). This analysis resulted in the identification of 30 significant KEGG pathways with a *p* < 0.05 (Table S4). Most of these pathways were found to be directly or indirectly involved in tumorigenesis. The Wnt/β‐catenin signalling pathway, known to be involved in cell proliferation and metastasis in various cancers [29, 30, 31, 32, 33], was enriched significantly by *Hoga1*‐coexpressed genes (Figure 4A). The regulation of β‐catenin phosphorylation is the central question in canonical Wnt/β‐catenin transduction [34], with increased β‐catenin phosphorylation occurring in the Wnt/β‐catenin pathway and tumour proliferation and metastasis being inhibited. There is evidence that the phosphorylation of β‐catenin (Thr41, Ser33 and Ser37) is dependent on the regulation of glycogen synthase kinase 3 beta (GSK‐3β) [35]. Our results strongly suggest that HOGA1 is an antioncogene in the kidney and regulates ccRCC via this pathway. To confirm this possibility, HOGA1 was expressed in the ccRCC cell lines 786‐O and 769‐P. Western blot results showed that the phosphorylation of β‐catenin at Thr41, Ser33 and Ser37 (have been reported to promote β‐catenin degradation) and the expression of GSK‐3β were increased significantly (Figure 4B). This suggested that the Wnt/β‐catenin signalling pathway was inhibited. Similarly, the Wnt/β‐catenin signalling pathway was activated in the HOGA1 knocked‐down HEK293T cells (Figure 4C). To further validate whether HOGA1 suppressed the growth and migration of ccRCC by inhibiting the Wnt/β‐catenin pathway, we transfected HOGA1 into the siRNAs against β‐catenin ccRCC cells. Overexpression of HOGA1 did not further inhibit cancer cell proliferation, invasion and migration in the β‐catenin knocked‐down ccRCC cell lines (Figure 4D–I). Given that c‐Myc and CyclinD1 are well‐established target genes of the β‐catenin/TCF transcription factor complex, we detected the two downstream factors of Wnt/β‐catenin in cells overexpressing HOGA1 with or without the silencing of β‐catenin. The results showed that HOGA1 overexpression decreased c‐Myc and CyclinD1 protein levels but did not alter the expression of c‐Myc and CyclinD1 in cells with knocked‐down β‐catenin (Figure 4J). These results collectively support the existence of a HOGA1–Wnt/β‐catenin–regulating pathway that inhibits ccRCC growth and metastasis.

### 3.5 HOG Promotes Wnt/β‐Catenin Signalling Pathway

**FIGURE 5:**
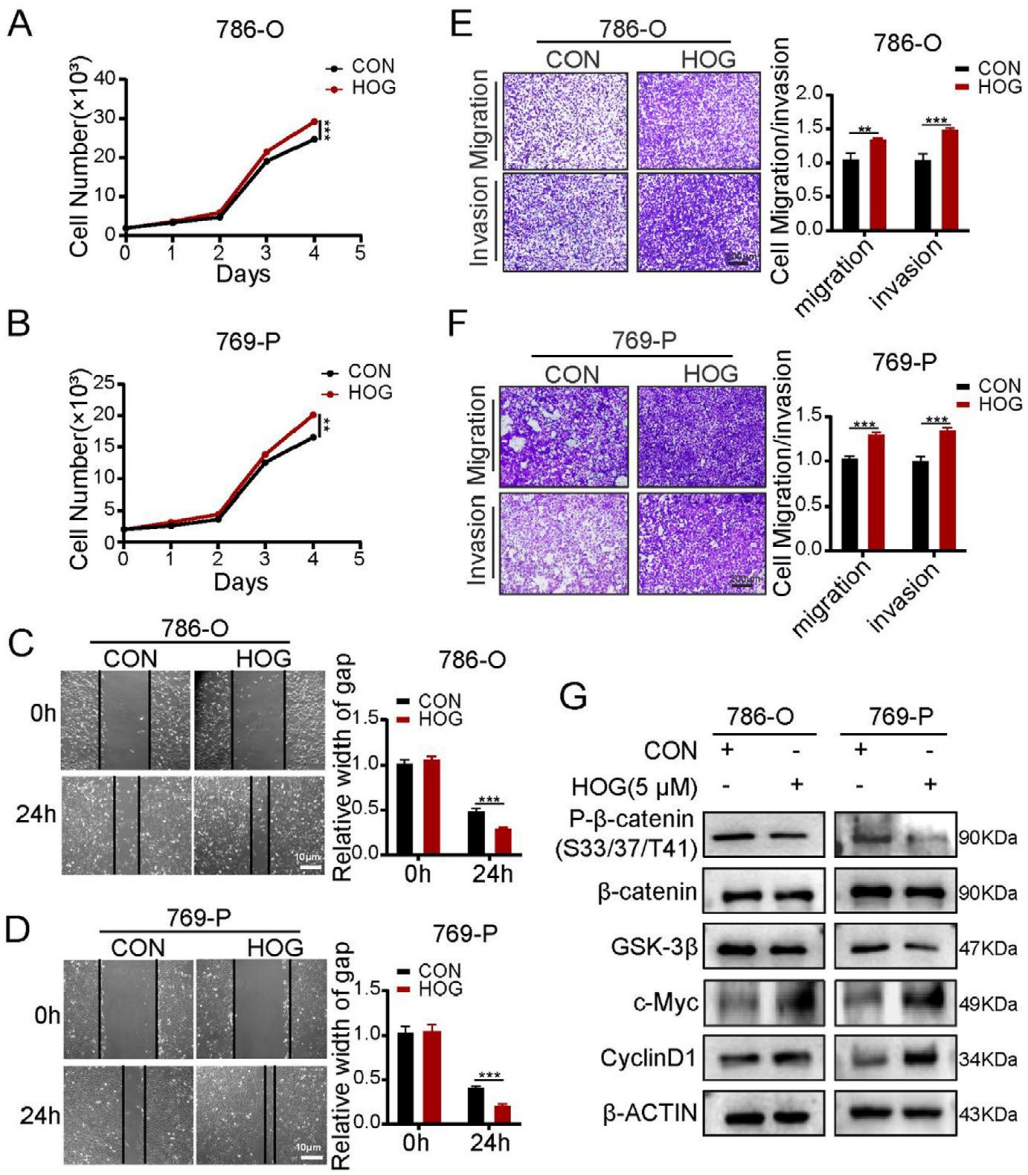
HOG promotes Wnt/β‐catenin signalling pathway. (A and B) Cell proliferation by CCK‐8 assay was validated in 786‐O and 769‐P cell lines. (C and D) Wound‐healing assay showed the migration ability of HOG. Scale bars: 10 μm. (E and F) Transwell assay was performed to verify the cell migration and invasion. Scale bars: 200 μm. (G) Western blot validated the effect of HOG on Wnt/β‐catenin signalling pathway. **p < 0.01, ***p < 0.001.

Changes in metabolic pathways in tumour cells are often accompanied by the accumulation of tumour‐related metabolites, which play an important role in tumour development [36, 37]. Our previous studies showed that HOGA1 was downregulated in expression in kidney cancer. The loss of metabolic enzymes is often accompanied by the accumulation of metabolic intermediates that are the catalytic substrate HOG. We therefore tested the effect of HOG in ccRCC at the cellular level and showed that HOG significantly promoted the proliferation, invasion and migration of ccRCC cells (Figure 5A–F). We also demonstrated that the accumulation of HOG activated the Wnt/β‐catenin signalling pathway (Figure 5G), although the exact mechanism of the activation requires further investigation.

### 3.6 HOGA1 Suppresses ccRCC Growth In Vivo

**FIGURE 6:**
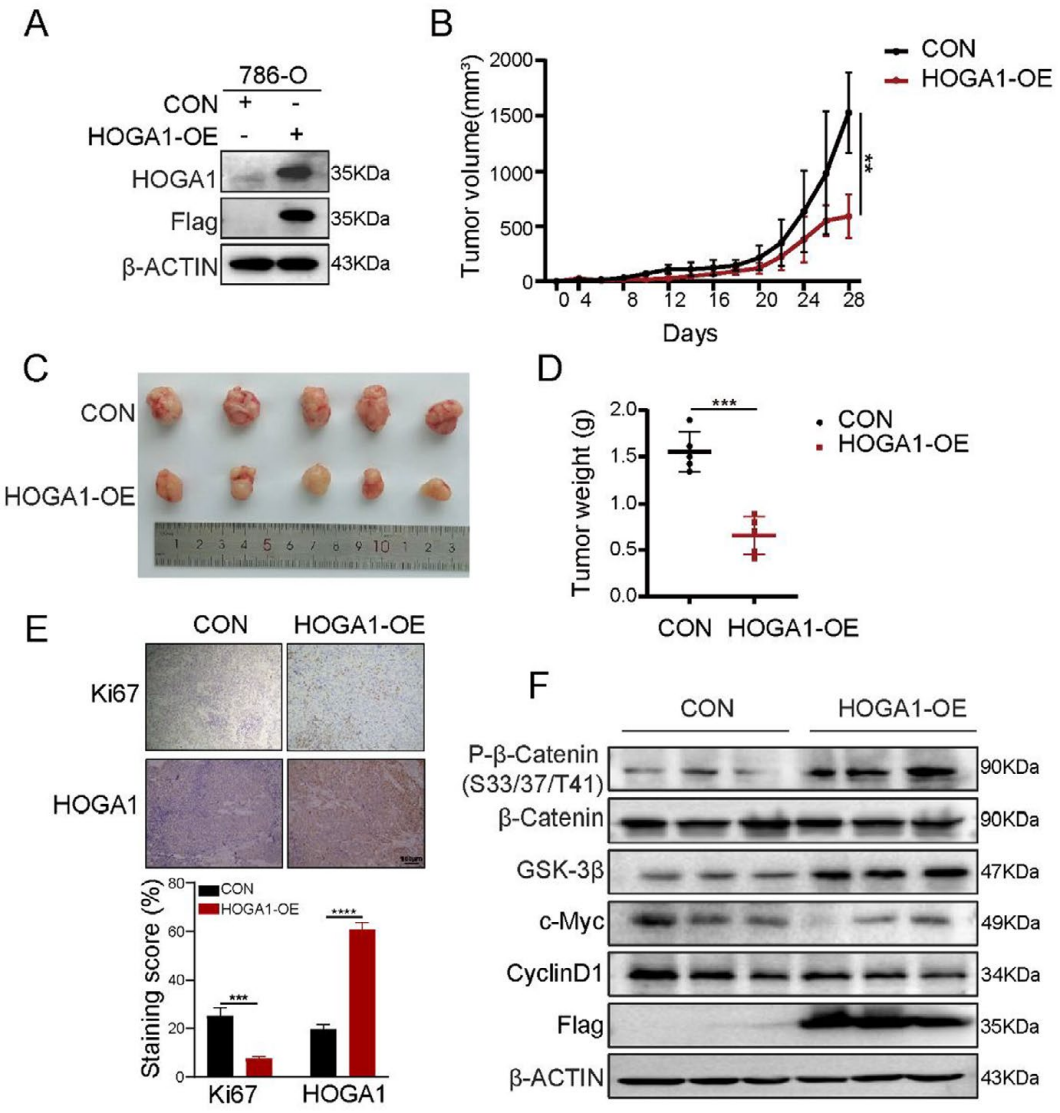
HOGA1 suppresses ccRCC growth in vivo. (A) HOGA1 protein expression in CON or HOGA1‐OE ccRCC cells. (B) 786‐O cells stably expressing HOGA1 were injected subcutaneously into 5‐week‐old male NOD SCID mice (*n* = 5), and growth curves of subcutaneous tumours were drawn in NOD SCID mice (*n* = 5). (C) The tumour size in two groups was shown in NOD SCID mice (*n* = 5). (D) The tumour weight in CON and HOGA1 groups were shown (*n* = 5). (E) Immunohistochemistry of HOGA1 and Ki67 and quantification. Scale bar: 100 μm. (F) Effect of overexpression of HOGA1 on Wnt/β‐catenin signalling pathway in vivo. **p < 0.01, ***p < 0.001 and ****p < 0.0001.

To further confirm the role of HOGA1 in ccRCC development in vivo, we established a tumour xenograft model in NOD SCID mice by subcutaneously injecting control or 786‐O cells with stable overexpression of HOGA1. We showed that the proliferation rate in the HOGA1‐OE group was significantly lower than that in the control group, while the subcutaneous tumour weight of the HOGA1‐OE group decreased significantly (Figure 6A–D). Staining of the cell proliferation marker Ki67 also showed a sharp decrease along with an increase in HOGA1 staining in tumour tissues (Figure 6E). Our results also demonstrated that overexpression of HOGA1 suppressed the Wnt/β‐catenin–c‐Myc/CyclinD1 axis (Figure 6F). Taken together, these results indicate that HOGA1 inhibits ccRCC proliferation via the Wnt/β‐catenin–regulating pathway in vivo.

## 4 Discussion

The exploration of metabolic reprogramming in cancer cells has become an increasingly prominent field within cancer research. Hydroxyproline metabolism, as one of the important metabolic processes in cells, has been found to accumulate hydroxyproline in liver cancer, which can promote HCC tumour progression and sorafenib resistance by regulating HIF1α [12]. Hydroxyproline can enhance INF‐γ‐induced PD‐L1 expression, inhibit autophagy and participate in the immunosuppression of the tumour microenvironment [38]. The role of hydroxyproline metabolism in tumours, especially kidney cancer, is far from being explored.

In this study, we found that HOGA1, which catalyses the final step of mitochondrial hydroxyproline metabolism from HOG to glyoxylate and pyruvate, had low expression in ccRCC through a proteomics analysis. The GEPIA database showed that low expression of HOGA1 was related to a worse overall survival rate in ccRCC patients. HOGA1 was reduced significantly in ccRCC, suggesting that it suppressed tumour gene function. To investigate the molecular mechanism by which HOGA1 inhibited ccRCC, we analysed the BXD mouse data in the GeneNetwork database. *Hoga1* is genetically regulated and is *cis*‐regulated, meaning that the gene locus for *Hoga1* is no longer regulated by other genetic factors. We therefore focused on finding the downstream regulatory network of *Hoga1*. Related gene searches and enrichment analysis showed that HOGA1 is involved widely in the regulation of ccRCC, with the most significant change occurring in the Wnt signalling pathway. Wnt/β‐catenin signalling is a key pathway in embryonic development and has been defined as one of the most important factors in tumorigenesis [39]. The canonical Wnt/β‐catenin signalling pathway operates by regulating the phosphorylation and degradation of the transcriptional cofactor β‐catenin and is controlled tightly by a complex of proteins including GSK‐3β [40]. KEGG pathway analysis showed that HOGA1 was enriched significantly in the Wnt/β‐catenin signalling pathway. To investigate the molecular mechanism underlying this association, we performed Western blot analysis and showed that overexpression and knock down of HOGA1 led to activation and inhibition of GSK‐3β and β‐catenin phosphorylation. It has been shown that nonphosphorylated β‐catenin accumulates in the cytoplasm and then enters the nucleus to bind TCF/LEF transcription factors, which promote transcription of the downstream target genes c‐Myc and CyclinD1 [41]. The results of the current study also demonstrated that HOGA1 regulates the expression of CyclinD1 and c‐Myc by modulating Wnt/β‐catenin signalling. The above results therefore show that HOGA1 significantly regulates the Wnt/β‐catenin–c‐Myc/CyclinD1 axis in ccRCC.

HOGA1 is a protein located in mitochondria, although how it regulates the upstream signals of Wnt/β‐catenin located in the cell membrane and cytoplasm has been the subject of further research. Recent studies have suggested that altered metabolic pathways in tumour cells are often accompanied by the accumulation of tumour‐related metabolites. These metabolites can act as signalling molecules [42], causing metabolism‐independent changes in tumour signalling pathways, thereby affecting tumour development. Tong et al. demonstrated that HOGA1‐targeting LARP7 could mitigate the tumour‐promoting effects of HOGA1 loss without disrupting hydroxyproline to glyoxylate metabolism [18]. However, this study did not explore the role of HOG, an intermediate metabolite in the hydroxyproline to glyoxylate metabolism. Recent studies have highlighted that elevated hydroxyproline levels may serve as a biomarker for monitoring the progression and treatment response of metastatic prostate cancer [43]. Additionally, urinary hydroxyproline excretion is closely linked to the diagnosis of various cancers [44]. Furthermore, some studies demonstrate that glyoxylate supplementation can alleviate the progression of colitis‐associated colon cancer by modulating metabolic pathways [45]. Therefore, it may be meaningful to explore the regulation of HOGA1 and HOG on hydroxyproline or glyoxylate metabolism in renal cancer in the future. At present, the role of HOG itself in cancer remains unclear. The experiments in the current study provide preliminary evidence that HOG has a role as a carcinogenic factor by promoting cell proliferation, migration and invasion. Pathway verification showed that HOG affects cell biological functions via the Wnt/β‐catenin signalling pathway. This suggests that HOGA1 deficiency, by causing the accumulation of the metabolite HOG, may affect the Wnt/β‐catenin signalling pathway, thereby regulating the occurrence and development of ccRCC. However, the potential interactions of HOGA1 with other key pathways in ccRCC, such as MAPK, P53 and PI3K/AKT—pathways known to be critical in tumorigenesis—remain to be explored [46, 47, 48]. This suggests that the role of HOGA1 and HOG in renal cancer may be more extensive and has yet to be fully explored. Therefore, a more comprehensive understanding of how HOGA1 may modulate these additional pathways could provide deeper insights into its role in ccRCC.

**FIGURE 7:**
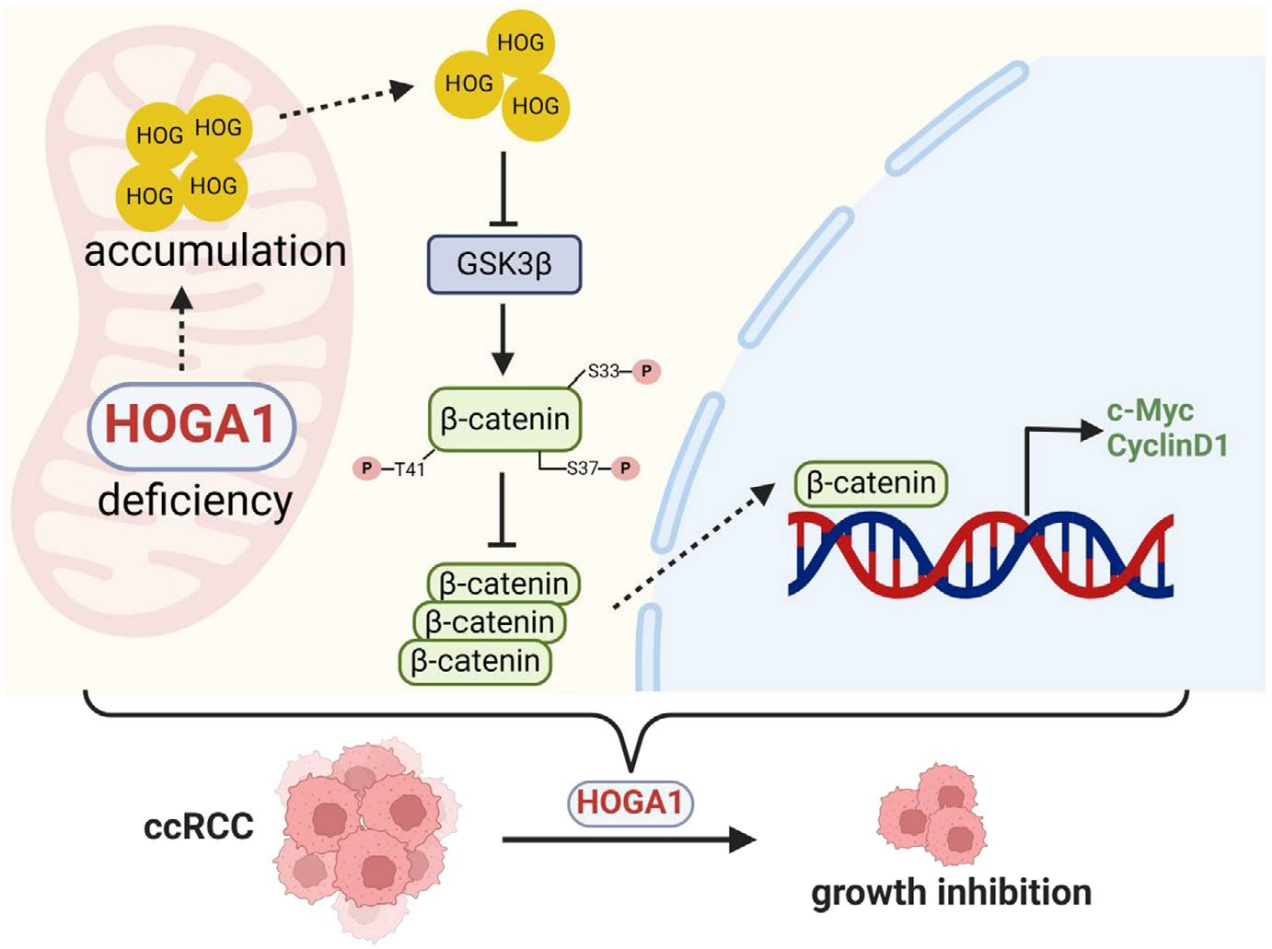
Schematic model for the mechanism and role of HOGA1 in ccRCC. The deletion of HOGA1 in ccRCC fosters the accumulation of HOG and suppresses the expression of GSK‐3β. This suppression results in a reduction in β‐catenin phosphorylation at Thr41, Ser33 and Ser37, thereby increasing β‐catenin accumulation in the Wnt signalling pathway. Consequently, an increased β‐catenin translocates to the nucleus, enhancing the expression of its downstream target genes, c‐Myc and CyclinD1, and ultimately facilitating the growth of ccRCC.

In summary, our current findings demonstrate that HOGA1 functions as a tumour suppressor in ccRCC, which can inhibit the Wnt/β‐catenin–c‐Myc/CyclinD1 axis by regulating the level of HOG (Figure 7), presenting a novel therapeutic strategy focusing on the regulation of hydroxyproline metabolism for ccRCC. Further research is, however, warranted on how HOG regulates the Wnt/β‐catenin signalling pathway and also influences the occurrence and development of kidney cancer.

## Author Contributions

Congmin Wang: data curation (equal), writing – original draft (equal). Yu Liu: conceptualization (equal), writing – review and editing (equal). Ying Tan: investigation (equal). Fuyi Xu: validation (equal). Mingyao Wang: investigation (equal). Yiming Tang: investigation (equal). Guofeng Nie: investigation (equal). Xiaodong Chi: resources (equal). Zhaowei Xu: formal analysis (equal). Yuxue Xu: supervision (equal). Baijiao An: formal analysis (equal). Geng Tian: funding acquisition (equal). Donglai Qi: funding acquisition (equal). Cuifang Yao: conceptualization (equal), data curation (equal), funding acquisition (equal), supervision (equal), writing – review and editing (equal).

## Ethics Statement

The study was conducted in accordance with the 1975 Declaration of Helsinki, and the project was approved by the Human Research Ethics Committee of Binzhou Medical College (Approval Code: 2021–202).

## Conflicts of Interest

The authors declare no conflicts of interest.

## Supporting information


**Table S1.** Clinical information of ccRCC patients.

**Table S2.** All proteins identified and quantified by proteomics.

**Table S3.** Differential proteins between clinical clear cell renal carcinoma tissues and normal tissues analysed by proteomics.

**Table S4.** All results for KEGG pathway analysis of genes related to *Hoga1* (*p* < 0.05).

**Figure S1.** Expression of HOGA1 in ccRCC tissues.
**Figure S2.** Role of HOGA1 in ccRCC in vitro.

## Data Availability

The data that supports the findings of this study are available in the Supporting Information of this article.
