# Supplementary material for: HOGA1 Suppresses Renal Cell Carcinoma Growth via Inhibiting the Wnt/β‐Catenin Signalling Pathway
Source: J Cell Mol Med. 2025 Mar 18;29(6):e70490. doi: 10.1111/jcmm.70490 (PMC11917137; doi:10.1111/jcmm.70490)
Supplement: Supplementary file 5 — Figure S1. Expression of HOGA1 in ccRCC tissues. Figure S2. Role of HOGA1 in ccRCC in vitro. [file JCMM-29-e70490-s002.docx]

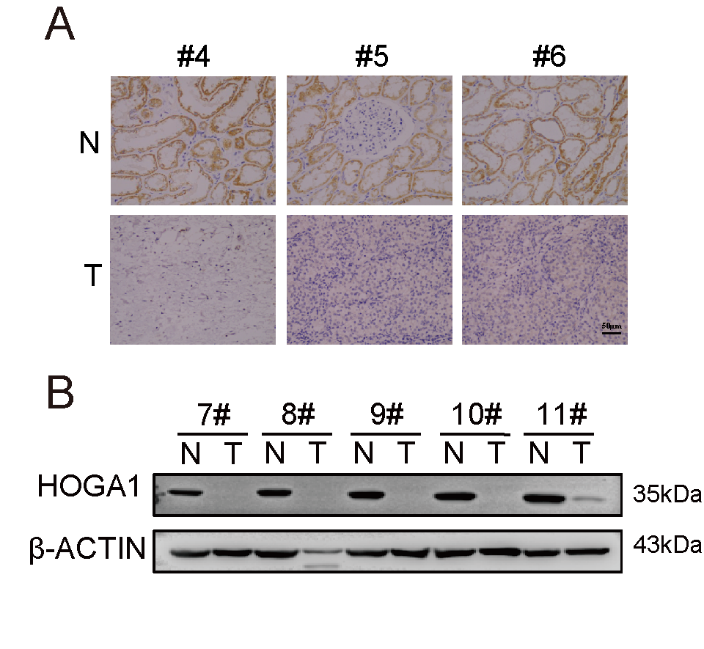
**Supplementary figure 1**

Expression of HOGA1 in ccRCC tissues. (A) Relative HOGA1 expression was detected by IHC between ccRCC tumors and adjacent non-tumor kidney tissues (4N/T-6N/T, 50μM). (B) Protein levels of HOGA1 in ccRCC tumors and matched adjacent non-cancer tissues were analyzed by western blot (7N/T-11N/T, n=5).

**
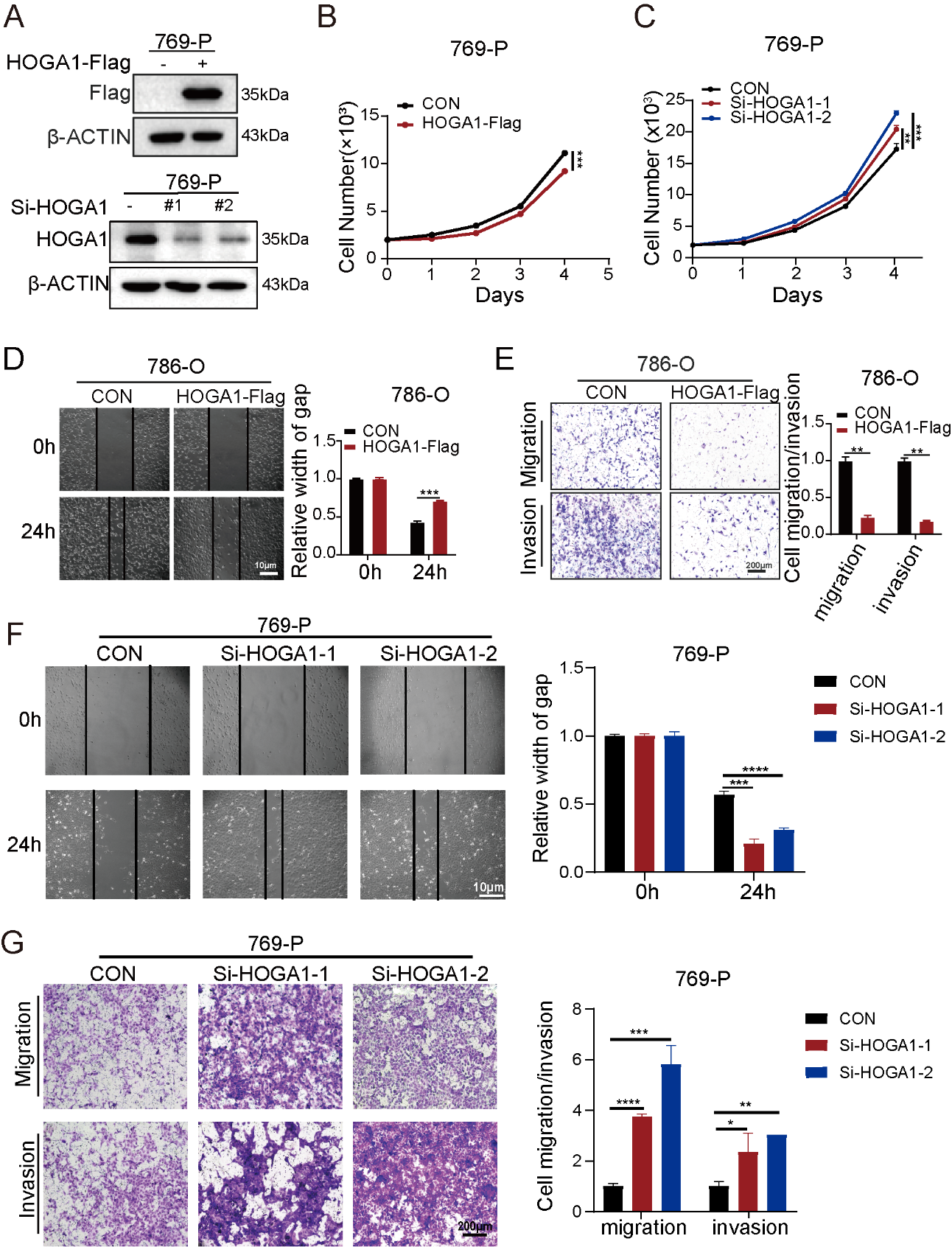
Supplementary figure 2**

**Role of HOGA1 in ccRCC in vitro.** (**A**) Western blot validated the expression of HOGA1 and HOGA1 siRNA transfection in 769-P cells. (**B-C**) CCK-8 assay were performed to examine the effect of HOGA1 overexpression and HOGA1 knockdown on cell viability in 769-P cells. (**D**) Wound healing assay determined the migratory distances of HOGA1 overexpression and control. Scale bars, 10 μm. (**E**) The effect of HOGA1 overexpression on cell migration and invasion was evaluated by transwell assay. Scale bars, 200µm. (**F**) Wound healing assay determined the migratory distances of HOGA1 knockdown and control. Scale bars, 10 μm. (**G**) The effect of HOGA1 knockdown on cell migration and invasion was evaluated by transwell assay. Scale bars, 200 µm. * *p* < 0.05, ** *p* < 0.01, *** *p* < 0.001, **** *p* < 0.0001.
